# A case report of Proteus syndrome (PS)

**DOI:** 10.1186/s12881-020-0949-x

**Published:** 2020-01-21

**Authors:** Xiaoyun Zeng, Xiaoming Wen, Xinxin Liang, Lina Wang, Lingling Xu

**Affiliations:** Department of Endocrinology, ShenZhen Hospital, Southern Medical University, No. 1333, Xinhu Road, Shenzhen, 518100 Guangdong China

**Keywords:** Proteus syndrome (PS), Case report, Literature reviewing, Milk coffee spots, Lower extremities with unequal length

## Abstract

**Background:**

Proteus syndrome (PS) is an extremely rare disease characterized by excessive chimeric growth of cells, and progressive and irregular asymmetrical hyperplasia.

**Case presentation:**

Herein, a PS case with atypical clinical features and syndromes was reported, to improve the understanding of the diagnosis and treatment of the disease. The case was a 3-year-and-11-month-old male child. He was admitted due to a primary diagnosis of McCune-Albright syndrome. After admission, the lesion samples from the milk coffee spots, and nodular thickening skin at hands and feet were subjected to genetic screening. Genetic testing results confirmed the diagnosis of PS.

**Conclusions:**

Based on the clinical manifestations, laboratory tests, imaging data, and literature reviewing, the etiology, diagnosis, treatment and prognosis of PS have been analyzed and discussed.

## Background

Proteus syndrome (PS), also known as Deformation syndrome, is a rare and sporadic congenital disease with asymmetrically and irregularly growing tissues. The asymmetric hyperplasia can be found in any tissue in the body, especially the skin, bone and adipose tissues. There have been around 100 cases of PS reported up to now, with the male -to-female rate of 1.9:1 [1, 2]. PS was first described and studied by Cohen and Hayden in 1979, which was named after Proteus in 1983 [3]. In 2011, it has been found that the gene mutation of PS is a serine/threonine protein kinase 1 (AKT-1) activating mutation in the chimeric cells. PS cases are rarely seen in clinic, with diverse and non-specific clinical manifestations, leading to the lack of understanding of clinicians, and the consequent misdiagnosis. Herein, we reported the clinical data analysis of a PS case diagnosed in our hospital. In combination with the literature review, this study aimed to obtain a comprehensive and in-depth understanding of the clinical features of PS.

## Case presentation

**Fig. 1:**
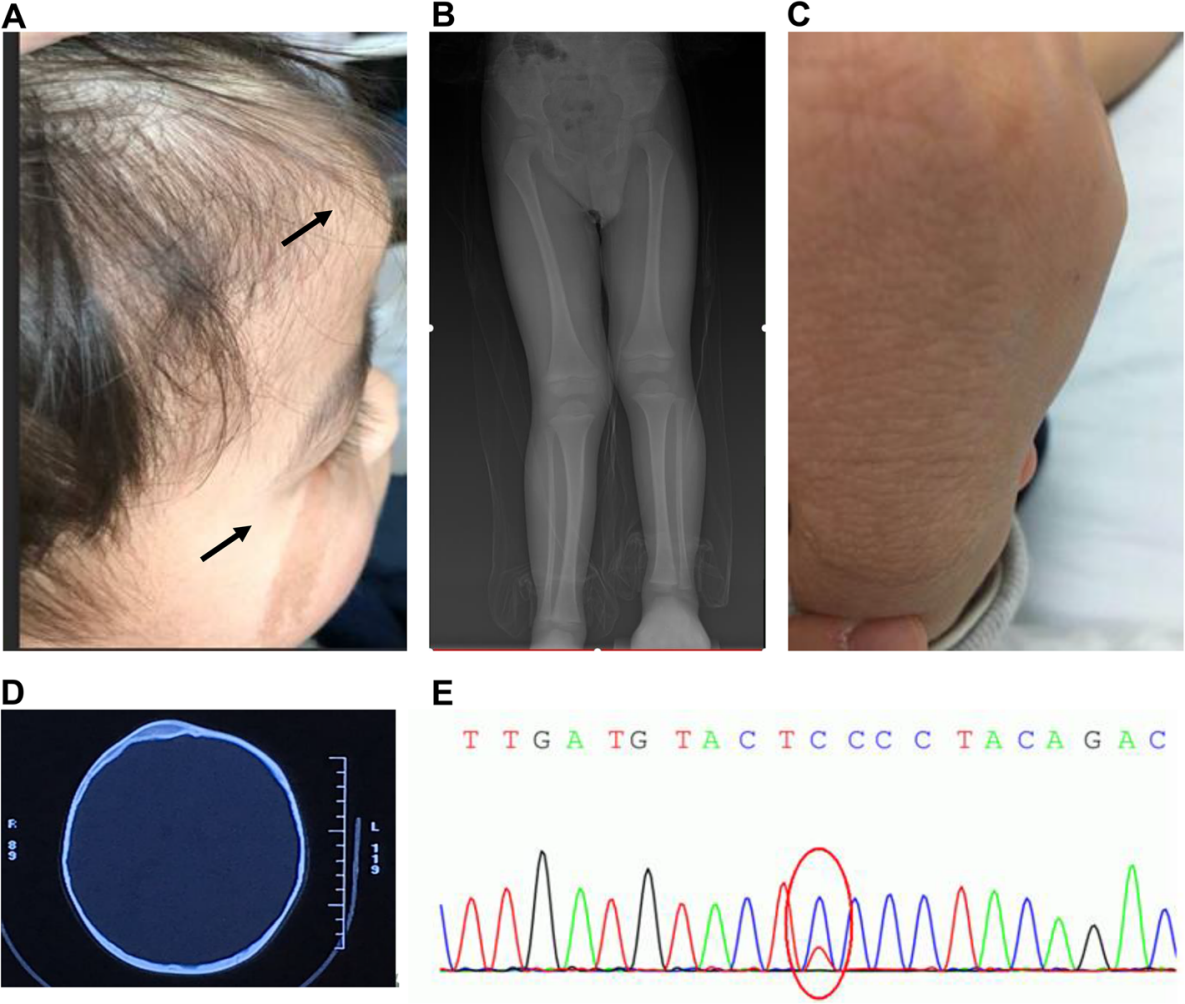
Representative images of the patient. **a** Milk coffee spots on the right facial skin (arrow) and right frontal bone bulging (arrow). **b** X-ray detection: the right lower extremity was longer than the left one. **c** Nodular thickening skin on right hand back. **d** Head CT detection: thickening of the local diploic space on the right side of the frontal bone, showing a glass-like density. **e** Disease gene screening of the lesion samples form the milk coffee spots, and nodular thickening skin at hands and feet. There was a heterozygous point mutation 49G > A (p.Glu17Lys) in the exon region of the AKT1 gene

This case was a 3-year-and-11-month-old male child, with no obvious abnormality at birth. However, the milk coffee spot was noted at the right face (Fig. 1a) at 2 month old, which was not treated. At the age of 1 year, the right frontal bulge was observed (Fig. 1a), which was gradually increased. After admission to the hospital, the head CT scan showed that, the local diploic space on the right side of the frontal bone was thickened, showing a glass-like density. Based on these detection results, the child was diagnosed as abnormal skull proliferation, and the skull replacement surgery after adulthood was recommended. At the age of 2 years, the length of lower extremities was found to be unequal. The X-ray showed that the right lower extremity was longer than the left lower extremity (Fig. 1b), and the correction with appropriate insole was recommended then. During 2016–2019, the head CT examination showed that the thickening range of the local diploic space on the right side of the frontal bone was enlarged, and the X-ray showed that the unequal length of the lower extremities increased from 2.9 cm to 3.7 cm. On January 4, 2019, in another hospital, the child received the osteophyte blocking at the distal right femur and proximal right tibia. Thereafter, the child was admitted to our hospital for the suspected McCune-Albright syndrome (MAS). Physical examination findings were as follows: height, 95.0 cm; and weight, 14.1 kg. Moreover, the right frontal bone bulging, milk coffee spot on the right face, local bulging at the midline of the right rib, and flaky nodules on the right hand and right thigh were observed (Fig. 1c). Furthermore, the right lower extremity was about 3.5 cm longer than the left lower extremity.

According to the auxiliary examinations, normal findings were obtained from the blood and urine routine tests, the liver and kidney function assessments, and the blood electrolyte and bone metabolism detections. The growth hormone (GH) and insulin-like growth factor-1 (IGF-1) were within the normal range. The sex hormone levels were as follows: estradiol (E2), < 5.00 pg/ml; luteinizing hormone (LH), 0.16 mIU/ml; follicle stimulating hormone (FSH), 0.58 mIU/ml; prolactin (PRL), 11.33 ng/ml; testosterone (Testo), < 0.03 ng/ml; 8 am cortisol, 472.00 nmol/L (133–537 nmol/L); and 8 am adrenocorticotropic hormone (ACTH), 5.15 pmol/L (1.6–13.9 pmol/L). The case was with normal thyroid function, negative for thyrotrophin receptor antibody (TRAb), thyroid peroxidase antibody (TPOAb), and thyroglobulin antibody (TgAb). The head CT detection showed local diploic space thickening on the right side of the frontal bone, with frosted glass-like density shadow. Superior orbital fissure and inferior orbital fissure were observed, while the optic nerve canal was not widened. Moreover, the accessory structures such as optic nerve and extraocular muscle were normal. Based on these findings, the bone fiber abnormal proliferation syndrome and osteofibroma had been considered (Fig. 1d). The X-ray showed that, the lower extremities were unequal in length, and the right lower extremity was obviously longer than the left one. There were no obvious abnormalities in the morphology, structure and density of the bilateral femur and tibia. Moreover, no abnormalities were found in the surrounding soft tissues. Ultrasound detection indicated that, the right rectus abdominis (8 mm in thickness) was significantly thicker than the left side (about 5 mm). For the analysis of the bone age, the head and hook bones were enlarged, and the fifth metacarpal ossification center was visible. The dermatoscopy indicated mild papillary hyperplasia. The skin CT detection showed hyperplasia at the lesion, with thickened epidermis, and papillary hyperplasia. Biopsy was performed based on the samples of the milk coffee spots, and the thickened nodular skin from the hands and feet, which were subjected to the DNA extraction and detection of disease-causing genes. The results showed that, there was a heterozygous point mutation 49G > A (p.Glu17Lys) in the exon region of the AKT1 gene (Fig. 1e). In silico algorithms including SIFT (sift.bii.a-star.edu.sg/), PolyPhen-2 (genetics.bwh.harvard.edu/pph2/), and Mutation Taster (www.mutationtaster.org/) were performed to predict this variation, and the predictive values were 0.01, 1.00 and 0.999 respectively, suggesting the pathogenicity of this mutation. Genetic testing results confirmed the diagnosis of PS.

## Discussion and conclusions

PS is a rarely seen asymmetrical and progressive histological hamartomatosis syndrome, with the estimated incidence of less than one in a million. The clinical manifestations of PS are diverse, and the imaging findings are complex and lack specificity, which may lead to clinical misdiagnosis or missed diagnosis. Certain diagnostic criteria for PS have been proposed, among which the relatively more comprehensive one is the primary and secondary diagnostic criteria from Turner et al. [4]. The primary main criteria include: (1) the chimerically distributed lesions; (2) the progressive disease course; and (3) the scattered distribution in the population. The secondary criteria include the following three categories: Class A, with the cerebral gyrus-like connective tissue nevus; Class B, including (1) the epidermal ridge (epidermal sputum/sebum adenine); (2) the disproportionate overgrowth for at least one of the following lesions: extremities, upper/lower extremity, hand/foot, and finger/toe; skull, bone hypertrophy; external auditory canal, bone hypertrophy; spinal dysplasia; and visceral lesions, spleen/thymus; and (3) specific tumors under 20 years of age; and Class C, including (1) the irregular distribution of adipose tissue: lipoma/local fat loss; (2) the vascular malformation: capillary/venous/lymphatic malformation; (3) pulmonary cyst; and (4) facial phenotype. PS cases should meet all three of the primary criteria, together with two of the Class A or B secondary criteria, or three of the Class C secondary criteria.

The earliest clinical manifestation of the case reported herein was the facial milk coffee spots. Skin milk coffee spots have an incidence of about 10% in normal population, which are however more commonly seen in NF1, MAS, and Waston syndromes. On the other hand, the skin characteristic changes in PS mainly include the cerebral gyrus-like connective tissue nevus, and linear verrucous nevus. In 1980s, Cohen et al. considered that PS did not show milk coffee spots. However, in 2003, Dragieva et al. [5] reported a case of PS patients with milk coffee spots. Moreover, in 2013, El-Hassani et al. [6] reported a 10-year-old PS child case with skin milk coffee spots. However, at present, there are no cases of PS with milk coffee spots reported in China. In this case report, the biopsy of the milk coffee spot skin of the child was subjected to the genetic test, and the results showed a heterozygous point mutation in the exon region of the AKT1 gene. However, more in-depths studies are still needed to confirm whether the milk coffee spots are one of the clinical manifestations of PS.

Nodular thickening was observed in the child’s extremity skin, which did not meet the diagnostic criteria for Class A (the cerebral gyrus-like connective tissue nevus) [7] and class B (epidermal caries). However, the genetic test confirmed the diagnosis of PS. The patient’s skin hyperplasia might be an early lesion, which has not deteriorate to develop epidermis and cerebral gyrus-like connective tissue nevus. Rodenbeck et al. [8] have reported a case of a 2-year-old PS child, who also showed skin nodular thickening. Therefore, the diagnostic criteria in 2004 might not be complete, which do not include the early symptoms or others. Through the genetic diagnosis, these early clinical manifestations might be considered to serve as the diagnostic criteria, providing evidence for the disease diagnosis confirmation.

In the case reported herein, there were two manifestations of skeletal involvement: (1) the skull bulging and the ground glass-like changes on CT; and (2) the lower extremities were unequal in length, and X-ray indicated no abnormal bone in the affected extremity. Among these, the asymmetric overgrowth of the right lower extremity was in line with the clinical manifestations of PS [9]. However, the case was characterized by the irregular skull bulging, cortical ground glass-like changes (as indicated by the CT detection), and facial skin milk coffee spots, which needs to be distinguished from MAS. The MAS gene screening was performed based on the milk skin spots on the patient’s facial skin, and the results did not support the diagnosis of MAS.

AKT-1 is an oncogene encoding the serine protein kinase AKT. It is involved in cell proliferation and apoptosis through the mammalian rapamycin (MTOR) signaling pathway and may be one of the causes for PS overgrowth. Therefore, the molecular genetic testing can be considered if the patient does not meet clinical diagnostic criteria. The clinical manifestations of the case reported herein did not fully meet the clinical diagnostic criteria, but the molecular genetic test of the facial milk coffee spots and nodular thickening skin tissue confirmed the diagnosis of PS. Therefore, for the suspected patients like this, the detection of mutation in the AKT1 gene should be recommended, which might be beneficial to the early diagnosis and prenatal diagnosis, avoiding the misdiagnosis and missed diagnosis.

At present, PS still lacks effective treatment. The current treatments mainly aim to minimize the degree of disability and improve the patient’s quality of life, i.e., only the symptomatic treatment for patients with PS [9]. However, the identification of the AKT1 mutation provides new information and theoretical basis for the diagnosis and treatment of the disease, as well as a new path for the drug development. Miransertib is an inhibitor that potently inhibits AKT 1, 2 and 3 isoforms and has been previously evaluated only in adult oncology trials. In 2019, Keppler-Noreuil et al. [10] reported that Miransertib had a certain prospect for the treatment of PS. The minimum age of the subjects in the study was 12 year old. However, the case reported herein was only 3-year-and-9-month old, and there have been no treatment experience and report of AKT1 inhibitor Miransertib at this age. In addition to this study, there have been no clinical studies of treatments with other PS drugs. Currently, the child case reported herein has no effective PS treatment. Only the symptoms have been closely observed, and the case have been receiving the symptomatic treatments.

## Data Availability

The datasets used and/or analyzed during the current study are available from the corresponding author on reasonable request.

## Abbreviations

ACTH: Adrenocorticotropic hormone
AKT1: Serine/threonine protein kinase 1
FSH: Follicle stimulating hormone
GH: Growth hormone
IGF-1: Insulin-like growth factor-1
LH: Luteinizing hormone
MAS: McCune-Albright syndrome
PRL: Prolactin
PS: Proteus syndrome
Testo: Testosterone
TgAb: Thyroglobulin antibody
TPOAb: Thyroid peroxidase antibody
TRAb: Thyrotrophin receptor antibody
